# Emerging Therapeutic Opportunities Based on Current Knowledge of Uveal Melanoma Biology

**DOI:** 10.3390/cancers11071019

**Published:** 2019-07-20

**Authors:** Raquel Vivet-Noguer, Malcy Tarin, Sergio Roman-Roman, Samar Alsafadi

**Affiliations:** Uveal Melanoma Translational Group, Department of Translational Research, Institut Curie, PSL Research University, 75248 Paris, France

**Keywords:** uveal melanoma, metastasis, targeted therapy, oncogenesis, Gαq pathway, BAP1, SF3B1, EIF1AX

## Abstract

Uveal Melanoma (UM) is a rare and malignant intraocular tumor with dismal prognosis. Despite the efficient control of the primary tumor by radiation or surgery, up to 50% of patients subsequently develop metastasis, mainly in the liver. Once the tumor has spread from the eye, the treatment is challenging and the median survival is only nine months. UM represents an intriguing model of oncogenesis that is characterized by a relatively homogeneous histopathological architecture and a low burden of genetic alterations, in contrast to other melanomas. UM is driven by recurrent activating mutations in Gαq pathway, which are associated with a second mutation in BRCA1 associated protein 1 (*BAP1*), splicing factor 3b subunit 1 (*SF3B1*), or eukaryotic translation initiation factor 1A X-linked (*EIF1AX*), occurring in an almost mutually exclusive manner. The monosomy of chromosome 3 is also a recurrent feature that is associated with high metastatic risk. These events driving UM oncogenesis have been thoroughly investigated over the last decade. However, no efficient related therapeutic strategies are yet available and the metastatic disease remains mostly incurable. Here, we review current knowledge regarding the molecular biology and the genetics of uveal melanoma and highlight the related therapeutic applications and perspectives.

## 1. Introduction

Uveal melanoma (UM) is the most frequent eye cancer in adults, representing 5% of all types of melanoma [1]. UM mainly arises from melanocytes within the choroid (85%), but it can also originate from the ciliary body (5–8%) or the iris (3–5%), to a lesser extent. The incidence of UM worldwide is estimated at 4.3 cases per million and it has remained stable for the last thirty years [1,2]. Uveal and cutaneous melanomas display major differences in the etiology, mutational profile, and clinical progression, despite sharing cell type and embryonic origin [3].

Uveal melanoma primary tumor can be effectively treated with radiation or surgical removal (enucleation) [4,5,6]. The prognosis of this cancer remains poor due to the development of metastases in 20–50% of patients, despite good local control [7]. These metastases mainly appear in the liver (89%) and they are particularly resistant to treatment, leading to an overall survival of six to twelve months. Current therapeutic approaches, including chemotherapies or targeted therapies, yield very low response rates (0–15%) in clinical trials, which highlights the need for more effective therapeutic strategies by identifying new targets or combined approaches [8,9].

## 2. Uveal Melanoma Risk and Prognostic Factors

Uveal melanoma risk factors consist of light skin and eye color (low pigmentation) [3]. UM mutation spectrum does not correlate with ultraviolet radiation (UVR) exposure [10,11,12], although UVR-induced mutational patterns (C-to-T transitions) have been described in rare cases (5.6%) [13]. Germline inactivating mutations in *BAP1* (BRCA1 associated protein 1) also represent a genetic risk factor in rare familial and bilateral UM cases, accounting for 2–5% of cases [14,15,16,17]. Recently, two UM cases have been reported to harbor germline loss-of-function mutations in *MBD4* (methyl-CpG binding domain 4) [18,19]. MBD4 plays a role in repairing DNA mismatches and its inactivation leads to a hypermutated tumor profile that is sensitive to immune checkpoint inhibitors [19,20].

The UM prognostic features include the age of the patient, tumor size, cell origin and heterogeneity, cytogenetic aberrations, and genetic profile [21,22,23,24]. No improvement in overall survival has been observed during the last 30 years, even though prognostication has improved due to the advances in understanding the genomic and genetic status of UM [25,26].

## 3. Biology-Based Therapeutic Strategies in Uveal Melanoma

### 3.1. Dysregulated Signaling Pathways

UM exhibits a dysregulation of a set of genes and pathways, most of which have been elucidated in the last two decades and that have been considered as candidates for therapeutic targeting. Here, we describe the potential therapeutic opportunities that are based on the main UM altered signaling pathways and related processes.

#### 3.1.1. Apoptosis and Cell Cycle

*BCL2* and *MDM2* are the first genes reported to be highly expressed in UM [27,28,29]. *TP53* is very rarely mutated, but is frequently inactivated by *MDM2* overexpression in UM. Consequently, Bcl2 and Mdm2 are described as potential targets for therapeutic intervention. For instance, treatment with inhibitors of the apoptotic proteins Bcl2/xL coupled with alkylating agents has been shown to trigger tumor growth inhibition in UM PDXs (Patient-Derived Xenografts) [30]. Clinical studies have failed to provide a therapeutic benefit due to strong adverse effects, although preclinical investigations of Bcl2 and Mdm2 inhibitors have confirmed their antitumorigenic effect in UM [30,31]. Evaluation of other strategies to re-activate p53, including inhibitors of Mdm4, a homolog of Mdm2, may offer good alternatives [32,33].

*Rb* (Retinoblastoma gene) inhibits proliferation and it is frequently inactivated in UM by phosphorylation induced by cyclin D1 (*CD1*) overexpression [34,35]. Precisely, CD1 is overexpressed in approximately 40% of cases [27,28]. In other cases, Rb phosphorylation may be due to p16^INK4a^ promoter methylation [36]. Rb pathway is disrupted in a wide number of cancers and the targeting approaches include CD inhibitors that are being tested in UM in combination with other therapies. HDAC (histone deacetylase) inhibitors are currently being assessed in UM and they have been found to induce CD1 degradation. Cotherapy with HDACi and CDKi has been shown to induce cell death in UM cell lines [32]. Additionally, CD1 activates CDK4/6, the downstream targets of the MEK pathway that is frequently altered in UM, which implies a potential co-targeting of MEK and CDK4/6.

#### 3.1.2. Hypoxia-Induced Response

*HIF* (Hypoxia Inducible Factor) is the main node for hypoxia response and it triggers a metabolic reprogramming when the growing tumors lack oxygen supply to increase glucose uptake and promote angiogenesis [37]. This hypoxia response occurs through cMET or CXCR4 (C-X-C chemokine receptor type 4). *HIF* is overexpressed in specific subsets of UM and its inhibition has been shown to suppress tumor growth in UM mouse models [10,38].

#### 3.1.3. cMET-PI3K Pathway

*cMET* encodes the transmembrane tyrosine kinase receptor that is activated through the binding of the hepatocyte growth factor (HGF). HGF is primarily produced in the liver and it is implicated in the growth of various malignancies. *cMET* expression levels are higher in UM metastatic tumors as compared to primary tumors, an intriguing fact given the presence of high levels of HGF in the liver tumor microenvironment. The HGF-cMET pathway has been described to mediate resistance to MEK inhibitors in metastatic UM [39]. In fact, HGF-cMET activates the PI3K-Akt pathway through PI3Kβ to compensate for the lack of MEK pathway activation. Therefore, blocking HGF-cMET signaling can resensitize the tumor cells to MEK inhibitors. This effect was observed in ex vivo UM metastatic explants [39]. A combination of MEK and cMET inhibitors is a promising approach that remains to be further investigated. On the other hand, the PI3K-Akt pathway is activated upon PTEN (Phosphatase and TENsin homolog) loss [40]. *PTEN* is a tumor suppressor that is underexpressed in 40% of UMs (mainly by LOH of the *PTEN* locus) [40,41,42]. There is growing evidence that PTEN is downregulated by miRNAs in UM [43,44]. Rescuing PTEN function is challenging but approaches targeting the PI3K/Akt pathway continue to be evaluated as combined therapies in UM.

#### 3.1.4. NF-κB Proinflammatory Signaling

NF-κB (nuclear factor-kappa B) pathway activation has been described to contribute to the mechanism of resistance to BET (Bromodomain and Extra-Terminal motif proteins) inhibitors in the UM cells. Inhibitors of NF-kB signaling synergized with BET inhibition in vitro and in vivo, which suggested that the inhibition of NF-kB signaling may improve the efficacy of BET inhibition in patients with advanced UM [45]. Furthermore, NF-κB signaling pathway contributes to *PRAME* (Preferentially Expressed Antigen in Melanoma) upregulation [46]. *PRAME* expression has been reported to correlate with the metastatic risk of UM [47]. These findings shed light on the potential targeting of this antigen by PRAME-specific HLA-A2 T-cell clones [48]. A recent study showed that 50% of metastatic UM expressed PRAME and HLA class I, which can be recognized by PRAME-specific T cells, implying the applicability of PRAME-TCR therapy on metastatic UM patients [49]. Currently, a PRAME-TCR clinical trial is ongoing for AML (Acute Myeloid Leukemia) and metastatic UM patients (NCT02743611).

### 3.2. Genomic Aberrations and Mutational Burden

**Figure 1:**
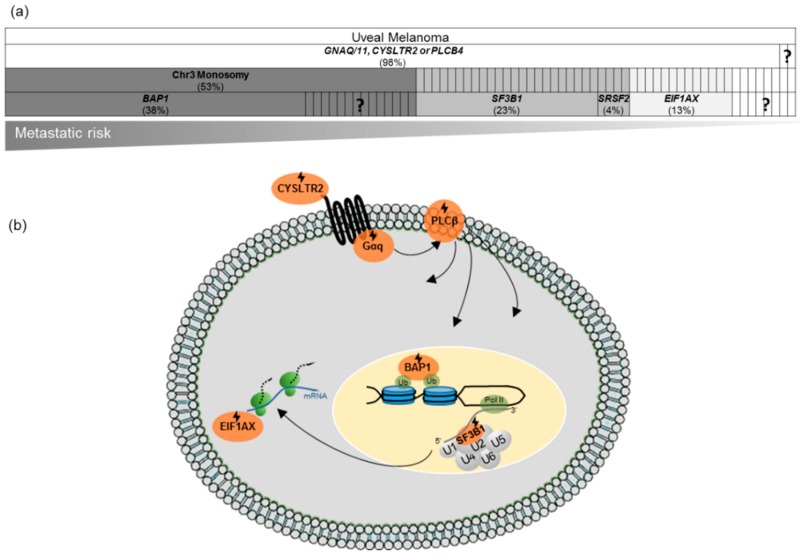
Genomic and genetic alterations in uveal melanoma (UM) and the affected biological processes. (**a**). Frequency of mutations in UM and the associated prognostic value: BRCA1 associated protein 1 (*BAP1)* mutations are mostly associated with chromosome 3 monosomy and an early metastatic risk (~5 years after primary UM diagnosis), splicing factor 3b subunit 1 (*SF3B1*) and serine/arginine-rich splicing factor 2 (*SRSF2*) mutations are mainly associated with chromosome 3 disomy and a late-onset metastatic risk (~8 years after primary UM diagnosis), while eukaryotic translation initiation factor 1A X-linked (*EIF1AX*) mutations are associated with chromosome 3 disomy and a low risk of metastasis. Data is retrieved from The Cancer Genome Atlas (TCGA) UM dataset (cBioportal for Cancer Genomics) [56,57]. (**b**). Main biological processes impacted by the recurrent mutations in UM. Mutations in components of G protein-coupled receptors (GPCRs) lead to the constitutive activation of Gαq signaling and several downstream pathways. Further oncogenic events include mutations in *BAP1*, *SF3B1/SRSF2*, or *EIF1AX*, involved in chromatin modulation, splicing, and translation initiation, respectively. Mutations are indicated by 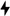.

Few genomic and genetic events characterize UM (Figure 1a). In fact, UM presents a low mutational burden, with an SNV mutation rate of <1 per Mb [11]. Additionally, UM displays a near-diploid karyotype with only a few chromosomal changes affecting chromosome 3 or chromosome arms 1p−, 6p+, 6q-, 8p−, 8q+. The cytogenetic alterations are tightly linked with the clinical outcome. The presence of both monosomy 3 and gain of 8q is correlated with high metastatic risk [50,51]. Harboring only one of the latter events correlates with an intermediate risk and the absence of such aberrations corresponds to a low risk of developing metastasis [10,11,50]. Notably, the monosomy of chromosome 3 is reported in up to 50% of primary UMs and it is considered to be a poor prognostic factor (Figure 1a) [17].

UMs are generally resistant to immunotherapy, which is probably due to their low mutational burden and the consequent low neoantigen generation. However, two exceptional UM cases have recently been reported to exhibit a response to PD-1 inhibitor. As previously stated, these cases are characterized by a hypermutated profile due to the presence of a germline loss-of-function mutation in *MBD4* [18,19].

### 3.3. Mutational Landscape and Related Therapeutic Perspectives

UM malignant transformation relies on two main events. First, a Gαq-pathway activating mutation in either *GNAQ, GNA11, CYSLTR2* (cysteinyl leukotriene receptor 2), or *PLCβ4* (phospholipase C *β*4), [13,52,53,54]. Second, a mutation in either *BAP1*, *SF3B1* (splicing factor 3b subunit 1), *SRSF2* (serine/arginine-rich splicing factor 2), or *EIF1AX* (eukaryotic translation initiation factor 1A X-linked) (Figure 1a,b) [10,55]. Based on the characterization of these genetic events, there is a growing interest in therapies targeting either Gαq downstream effectors, BAP1-related molecular mechanisms, splicing, or further related biological processes.

#### 3.3.1. Gαq-Pathway Activating Mutations

**Figure 2:**
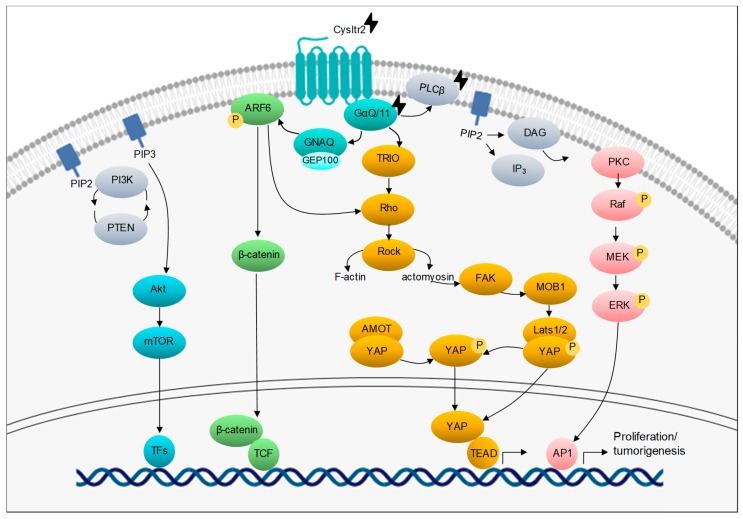
Dysregulated pathways in uveal melanoma. Recurrent mutations in *GNAQ*, *GNA11*, *PLCβ4,* and *CYSLTR2* are mutually exclusive and trigger the activation of Gαq signaling and related pathways (Akt/mTOR, Wnt/β-catenin, Yes-associated protein (YAP), and MAPK pathways) in UM. Mutations are indicated by 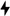.

The first UM driver event consists of mutations that activate the Gαq pathway [13,52]. *GNAQ/11* mutations are reported in approximately 96% of UM patients, mainly at codon Q209 and less recurrently at R183 or G48 [13,58]. *PLCβ4* and *CYSLTR2* mutations have been recently reported at lesser frequencies (2.5% and 4%, respectively) [10]. *PLCβ4* hotspot mutation is located at p.D630, the region corresponding to the phospholipase C β-4 catalytic domain [53], and *CYSLTR2* mutation encodes an L129 substitution [54]. These mutations are mutually exclusive hotspot mutations that activate the Gαq signaling, thereby stressing the importance of this pathway in UM oncogenesis (Figure 2) [53,54]. Of note, none of these mutations is correlated with differential prognosis or clinical outcome, which suggests an oncogenic rather than metastatic driver effect [10,54,59].

*GNAQ* and *GNA11* encode the subunits Gαq and Gα11 that are bound together with β and γ subunits. The resulting heterotrimeric complex is coupled with a GPCR protein (G protein-coupled receptor), which is involved in several signaling transduction pathways, as shown in Figure 2. In the basal state, Gαq/11 is bound to a GDP and it remains inactive. Upon GTP binding, the complex undergoes conformational changes and then targets downstream effectors [52]. *GNAQ*/*11* mutations lead to a constitutively active α subunit, which results in a dysregulation of several downstream pathways including Akt/mTOR, Wnt/β-catenin, Rac/Rho, MAPK, and PI3K pathways [60].

The importance of Gαq pathways in UM oncogenesis has been described in vitro and in vivo. Accordingly, *GNAQ/11* knockdown inhibits the growth of *GNAQ/11*-mutated UM cell lines, an effect that is not observed in *GNAQ/11* wild-type (WT) cell lines [61]. Moreover, mouse models that harbor *GNAQ*/*11* mutations develop multiple tumors, which confirms the oncogenic impact of these mutations [13,62,63]. Mice with melanocyte-specific expression of GNA11^Q209L^ recapitulated human Gq-associated melanomas and developed pigmented neoplastic lesions from the melanocytes of the skin and non-cutaneous organs, including the eye and leptomeninges, as well as atypical sites, such as the lymph nodes and lungs [62,63].

##### Gαq-Corresponding Therapeutic Strategies

Gαq/11 inhibitors development has been a major concern over the last two decades, given the high recurrence of *GNAQ* and *GNA11* mutations in UM. YM-254890 (YM) is a cyclic depsipeptide that is extracted from bacteria that acts as a selective Gαq inhibitor by preventing the GDP release, leading to the blockage of GDP/GTP exchange reaction and Gαq activation [64,65]. Interestingly, YM was shown to inhibit R183 Gαq mutant rather than Q209L Gαq mutant [65,66]. FR900359 (FR) is a YM analog that was obtained from plants that depicts a similar mode of action. FR has been recently described to trigger differentiation and inhibit the migration of *GNAQ/11*-mutated melanoma cells [67]. FR mainly inhibits Q209L, Q209P, and Q209L Gαq/11 mutants, promoting UM cell cycle arrest and cell death [68]. Despite the promising results of Gαq/11 inhibitors in vitro, such inhibitors have not yet been evaluated for clinical application.

On the other hand, much attention has been drawn on targeting the Gαq downstream effectors Protein Kinase C (PKC) and MEK. The inhibition of each of these pathways has been evaluated, but showed no clinical benefit, which suggested the need for combinatory strategies to abolish different Gαq downstream effectors at once [69,70]. The inhibitors of MEK and PI3K (MEKi, PI3Ki) separately show a modest apoptotic effect on *GNAQ/11*-mutated UM cell lines that is significantly increased upon combination [61,71,72]. Similarly, PI3Ki and mTORi exhibit an apoptotic effect in a wide range of UM cells and tumor growth inhibition in vivo [73]. Another promising strategy is coupling PKC inhibition with p53 activation. Cotreatment with Mdm2i and PKCi decreases the growth rate of the UM cells and promotes cell death that is induced by DNA damage [74]. In vivo studies show that the dual inhibition of PKC and Mdm2 or PKC and mTOR reduces tumor growth in UM PDXs [31]. These results have boosted the assessment of such compounds in clinical trials.

Recent findings pinpointed ARF6 as a downstream effector of Gαq [75]. Interestingly, ARF6 is a GTPase that is known to play a role in proliferation, invasion, and metastasis in some cancers [76,77]. In UM, inhibiting ARF6 induces a decrease in proliferation in vitro and tumorigenesis in vivo [75]. Moreover, activated ARF6 triggers the transport of β-catenin to the nucleus, where it can activate transcription factors, thereby promoting invasion and metastasis [75]. β-catenin is the main node in the canonical Wnt pathway, which plays a vital role in embryonic development and it is known to be mutated in various cancers [78]. β-catenin and its downstream effector Wnt5a were found to be overexpressed in a subset of aggressive UM tumors [79]. Moreover, β-catenin inhibition was shown to induce apoptosis and inhibit cell growth, invasion, and migration in vitro [80].

Hippo pathway, together with the mTOR (Mammalian Target of Rapamycin) pathway, regulate organ size in mammals [81]. YAP (Yes-associated protein) is one of the main effectors of the Hippo pathway, but it can also be activated in a Hippo-independent manner by Gαq through Trio-Rho/Rac or through MOB1 phosphorylation [82,83]. In proliferating cells, YAP is active until a certain cell density is reached. Subsequently, MTS1 and MTS2 (mammalian STE20-like protein kinase 1 and 2) activate LATS1/2 (large tumor suppressor homolog 1 and 2) that phosphorylate YAP, which will stay in the cytoplasm and be further degraded, which leads to growth inhibition [82,84]. On the contrary, dephosphorylated YAP remains in the nucleus, where it can bind to TEAD (transcriptional enhancer activation domain), inducing gene expression and eventually cell proliferation [81]. All the UM cell lines harboring *GNAQ/11* mutations exhibit low YAP phosphorylation and nuclear localization, which indicates YAP activation. The cell growth of *GNAQ/11*-mutated UM cells is significantly decreased upon *YAP* knockdown or inhibition [84,85]. Notably, a recent study identified GPCR-mediated YAP activation and RTK-driven AKT signaling as key pathways that are involved in the escape of UM cells from MEK inhibition [86]. Verteporfin is a drug that is used for the treatment of vascular occlusion of abnormal blood vessels and it has been reported to inhibit TEAD-YAP interaction [85,87]. However, its specificity to YAP has not been confirmed. In UM cells, verteporfin decreased colony formation and proliferation in three-dimensional (3D) cultures. Moreover, verteporfin reduces tumor size and cell proliferation in vivo [82,84]. Recently, FAK has been revealed to activate YAP by MOB1 phosphorylation, resulting in Hippo pathway inhibition. FAK inhibition has been shown to abolish YAP-dependent UM tumor growth in vitro and in vivo [83].

Overall, the successful inhibition of Gαq-signaling-dependent oncogenesis may be achieved by synergistically targeting several downstream effectors. Additional therapeutic strategies have to be pursued for the metastatic settings provided that *GNAQ/11* mutations have no prognostic value in UM.

#### 3.3.2. *BAP1*, *SF3B1*, *SRSF2* or *EIF1AX* Mutations

The second oncogenic event of UM consists of mutations in *BAP1, EIF1AX, SF3B1,* or *SRSF2.* These mutations are mutually exclusive in almost all UM cases [10,55,88]. *BAP1* mutations are recurrently found to be associated with chromosome 3 monosomy in early metastatic risk cases. Mutations on *SF3B1* and *SRSF2* are mainly associated with chromosome 3 disomy and a late-onset metastatic risk, while *EIF1AX* mutations are associated with chromosome 3 disomy and a low risk of metastasis (Figure 1a) [55,88].

##### *BAP1* 

*BAP1* encodes a deubiquitylase that forms protein complexes that are implicated in several pathways along with cell cycle, cell differentiation, and DNA damage response and it has been described to act as a tumor suppressor in various cancers [17,89,90]. The expression of *BAP1* is lost in up to 84% cases of metastatic UM, due to inactivating mutations. *BAP1* is mutated in 38% of primary UMs, mainly in tumors with monosomy 3, thereby being characteristic of belligerent tumors [10,15,91]. Remarkably, around 84–89% of metastatic tumors harbor somatic mutations in *BAP1*. Hence, *BAP1* alterations are strongly correlated with a higher metastatic risk and reduced survival rate [10,15,59,91,92]. Therefore, targeting BAP1-related processes represents a promising therapeutical strategy for preventing metastatic progression and improving patient survival. BAP1 binds to ASXL1 to form the polycomb complex that deubiquitinates histone 2A [93,94]. Thus, the loss of *BAP1* increases ubiquitinated expression and it may sensitize tumor cells to HDAC (histone deacetylase) inhibitors, like valproic acid, trichostatin A, LBH-589, and syberynalide hydroxamic acid. HDAC inhibition has been shown to stop cell proliferation, induce cell cycle arrest, trigger apoptosis, block migration, promote cell differentiation, and impact the gene expression profile in preclinical UM models [95,96,97]. A very recent study demonstrated that the combination of MEK and HDAC inhibitors considerably decreased tumor growth in both subcutaneous and liver metastasis xenograft models of UM, which encourages clinical co-targeting of MEK and HDAC in advanced UM [86].

EZH2 (Enhancer of Zeste Homolog 2) forms the polycomb repressive complex 2 (PRC2), which methylates histone H3 lysine 27 (H3K27). BAP1 loss leads to increased H3K27 that, in turn, raises the expression level of EZH2 [98]. However, the UM cells were reported to resist EZH2 inhibition regardless of their *BAP1* status [99].

Additionally, BAP1 forms a complex with BRCA1 and BARD1, which takes part in double-strand break repair through homologous recombination (HR) [100]. BAP1 deficiency results in impaired HR, which may suggest an increased dependency on other DNA repair pathways and a consequent sensitivity to PARP inhibition [100,101,102]. A clinical trial of a PARP inhibitor (Niraparib) in BAP1-deficient neoplasms including UM is ongoing (NCT03207347).

Overall, targeting BAP1-related processes is a potential therapeutic strategy. Nevertheless, successful approaches to target metastatic malignancies may require combined treatment in order to block all the related processes. A synthetic lethality screen can be a precious tool in revealing vulnerabilities to therapy in BAP1-deficient UM patients.

##### *EIF1AX* 

*EIF1AX* missense mutations are recurrent in 13% of UMs. These mutations are mainly associated with disomy 3 and present a low metastatic risk [10,88]. *EIF1AX* encodes eukaryotic translation initiation factor 1A (eIF1A) and it is essential in the recruitment of the ternary complex and for assembling the 43S preinitiation complex (PIC) [103]. Translation initiation is a rate-limited step that is tightly regulated and factors taking part at this stage are known to be misregulated in tumorigenesis [104]. *EIF1AX* overexpression has been documented to boost translation and cell proliferation in bovine mammary epithelial cells [105]. Interestingly, *EIF1AX* was found to harbor heterozygous mutations in papillary carcinomas, the most common thyroid cancer, and in ovarian carcinoma with a worse prognosis when coupled with mutations of the Ras family [106,107]. Very recently, *EIF1AX* and *RAS* mutations have been shown to cooperate to induce tumorigenesis in isogenic cell lines and mice. EIF1AX-A113splice variants, which are recurrent in advanced thyroid cancer, stabilize the PIC and enable a general increase in protein synthesis through ATF4-induced dephosphorylation of EIF2α. RAS stabilizes c-MYC, which cooperates with ATF4 to sensitize mTOR to amino acid supply. These combined events were shown to generate therapeutic vulnerabilities to MEK, BRD4, and mTOR kinase inhibitors [108]. These findings pinpoint new therapeutic strategies and emphasize the importance of understanding the biological impact of different *EIF1AX* mutations in UM.

##### *SF3B1* and *SRSF2*

The splicing factor (SF) genes *SF3B1, U2AF35*, *ZRSR2*, and *SRSF2* are recurrently mutated in hematological malignancies [109,110,111] and solid tumors [112,113,114], which include UM [11,88,115,116]. It is noteworthy that the SF hotspot mutations take place in a mutually exclusive manner and they affect proteins that are involved in the 3′ splice site (3′ss) recognition, an early step of splicing, resulting in specific aberrant splicing patterns. *SF3B1* and *SRSF2* mutations are recurrent in UMs and they lead to a change of function of the SF [109]. Such events highlight the involvement of splicing aberrations in oncogenesis and the relevance of SF therapeutic targeting.

*SRSF2* belongs to the family of serine/arginine (SR)-rich proteins that aid splicing through binding exonic splicing enhancers (ESEs). SR proteins contain at least an SR rich binding domain and an RNA recognition motif (RRM), where RNA binding proteins (RBPs) attach. On early steps of splicing, SF1 binds to the BP and SRSF2 and ZRSR2 simultaneously bind to ESEs to aid the binding and stability of the U2AF subunits [117]. *SRSF2* is most commonly mutated in chronic myelomonocytic leukemia (CMML) (47%) [118] and myelodysplastic syndromes (MDS) (15%) [119]. Recently, *SRSF2* has also been found to be mutated in 4% of UMs [10,120]. Upon hotspot mutations at P95m which is located downstream the RRM, SRSF2 undergoes a conformational change on the RRM, and consequently acquires more affinity for G-rich versus C-rich ESEs motifs, differently from the WT, which has an equal affinity for these motifs [109,120,121,122]. The resulting misregulated exon inclusion causes an aberrant splicing pattern of a broad range of genes comprising the tumor suppressor *ARMC10* or *EZH2* [122,123]. The mis-spliced form of *EZH2* is sensitive to nonsense-mediated RNA decay (NMD), which implies a decrease in EZH2 levels which has already been observed in MDS progression [124]. In fact, *EZH2* and *SRSF2* mutations take place in a mutually exclusive manner [122]. Even though the *SRSF2* mutation rate is low in UM [10,120], it may be a significant event, given the cascade effect of misrecognition of ESEs on a large number of target genes.

*SF3B1* encodes the U2 small nuclear riboprotein complex (U2-snRNP) that is responsible for branchpoint (BP) recognition and it is mutated in 23% of UMs [116]. U2-snRNP binds to the BP of the intron in an incomplete manner. Further interactions are required to enhance BP identification and stabilize the interaction, including base-pairing that is mediated by SF3B1 and U2AF35/65 binding. SF3B1 structure consists of a hydrophilic N-terminal harboring a U2AF-binding motif and a C-terminal with 22 different HEAT (Huntingtin, Elongation factor 3, protein phosphatase 2A, Targets of rapamycin 1) repeats [125,126], whose function remains to be elucidated [120]. Hotspot mutations target the HEAT repeats at codons R625, K666, and K700 [125,126]. Hotspot mutation K700 prevails in hematological malignancies, whereas the R625 and K666 mutations prevail in UM and they are frequently associated with disomy 3 and a late metastatic risk [11,55,88,115]. *SF3B1* mutations have been thoroughly investigated and were reported to induce an aberrant splicing pattern by an alternative 3′ss usage upstream the canonical 3′ss in breast cancer, CLL, and UM [116,127]. *SF3B1* mutations generate change-of-function mutants, leading to aberrant splicing of less than 1% of all splice junctions by recognizing an alternative BP localized at 11-14 nts upstream the canonical site [116]. SF3B1 has been reported to be involved in the splicing of key apoptotic genes, like MCL1 and BCL2/xL, which are appealing cancer targets [128]. Yet, further studies are required to link the splicing aberrations to oncogenesis. These findings have resulted in a growing interest in splicing modulators as therapeutic agents. Microbial and natural metabolites that inhibit splicing were the first candidates, including FR901464 and derivatives. The FR9014 series was isolated from Pseudomonas sp. Number 2663 and constitutes the first antiproliferative molecules that are associated with splicing inhibition. Spliceostatin A is a methylated derivative of FR901464. Spliceostatin B was also isolated from Pseudomonas sp. Number 2663. Spliceostatin E was isolated from Burkholderia sp. FERM BP3421. Thailanstatins were recovered from Burkholderia thailandensis MSMB43. Meayamycin and Sudemycins are synthetic derivatives from the depicted natural products [129,130]. These splicing inhibitors have been shown to regulate Mcl-1 splicing and inhibit cell proliferation in a dose-dependent manner [131,132]. Other compounds that were isolated from bacteria include Pladienolides A-G, E7107, FD-895, and herboxidiene. E7107 has been tested on clinical trials in various solid tumors. No significant response was observed, even though the mRNA levels were altered in a dose-dependent manner [133]. An additional natural compound that is extracted from plants, isogingketin, was also described as a general splicing inhibitor with anti-tumor activity [134]. Nevertheless, inhibiting an essential biological process, like splicing, confers high cytotoxic effects, thereby limiting the therapeutic window [135,136]. Specific compounds are then needed to restore the normal splicing level, rather than inhibiting the whole process of splicing.

Recently, encouraging results were obtained with H3B-8800, a small molecule that is derived from pladienolide that targets SF3B1 complex. Cells harboring *SF3B1* mutations presented higher sensitivity to this inhibitor than cells with WT *SF3B1*, a feature that may overcome the high cytotoxicity of splicing inhibition. The preferential inhibition is associated with an enrichment of alternative 3′ss in SF3B1 mutant cells as compared to WT cells [137]. Further studies are ongoing to confirm the specificity of H3B-8800 in vivo and in a clinical trial in patients with advanced myeloid malignancies, including MDS, AML, and CMML (NCT02841540).

New perspectives also emerged from the studies of neopeptides that were generated by the aberrant transcripts in *SF3B1*-mutant cells. In fact, the splicing-derived putative neoepitopes have a high degree of recurrence, which is suggestive of potential interest for immunotherapeutic intervention. Moreover, these neopeptides are considered for prospective personalized cancer vaccine development [138].

## 4. Conclusions

UM is a rare cancer in adults, with very stereotyped oncogenic events that have been mostly decrypted over the last 10 years. The epidemiological, genetic, and transcriptional specificity of UM highlight the importance of UM as a model of oncogenesis. The understanding of the molecular mechanisms that underlie UM has considerably progressed over the last decade. However, these advances have not yet been translated into therapeutic progress, and the prognosis of the metastatic form of UM remains somber.

Conclusively, targeted therapies remain to be improved by combinatory strategies in light of a better understanding of the UM-underlying molecular mechanisms. Recently-reported exceptional immune responses in UM patients harboring *MBD4* mutations point up the importance of deciphering cancer mechanisms in order to determine the oncogenic actors and develop the appropriate therapeutic strategies. Moreover, the development of preclinical models that recapitulate the different routes of UM malignant transformation is essential for validating novel therapeutic strategies.
